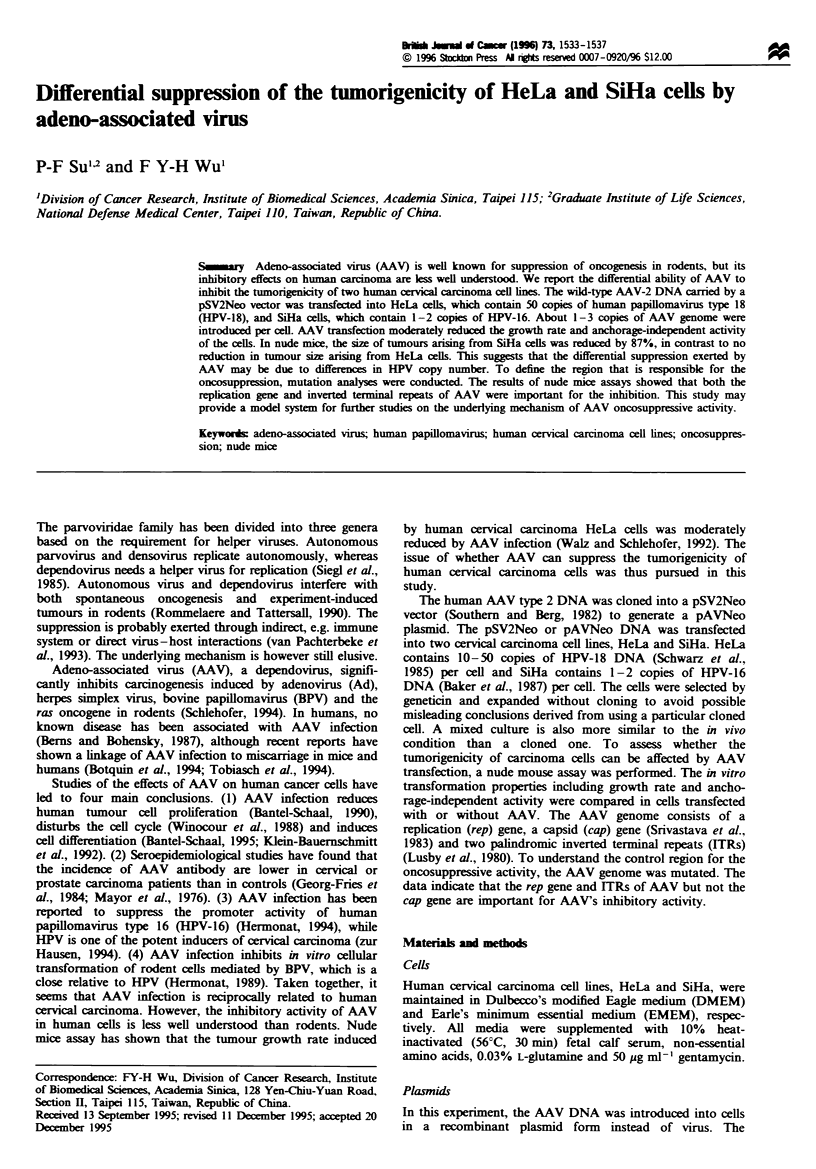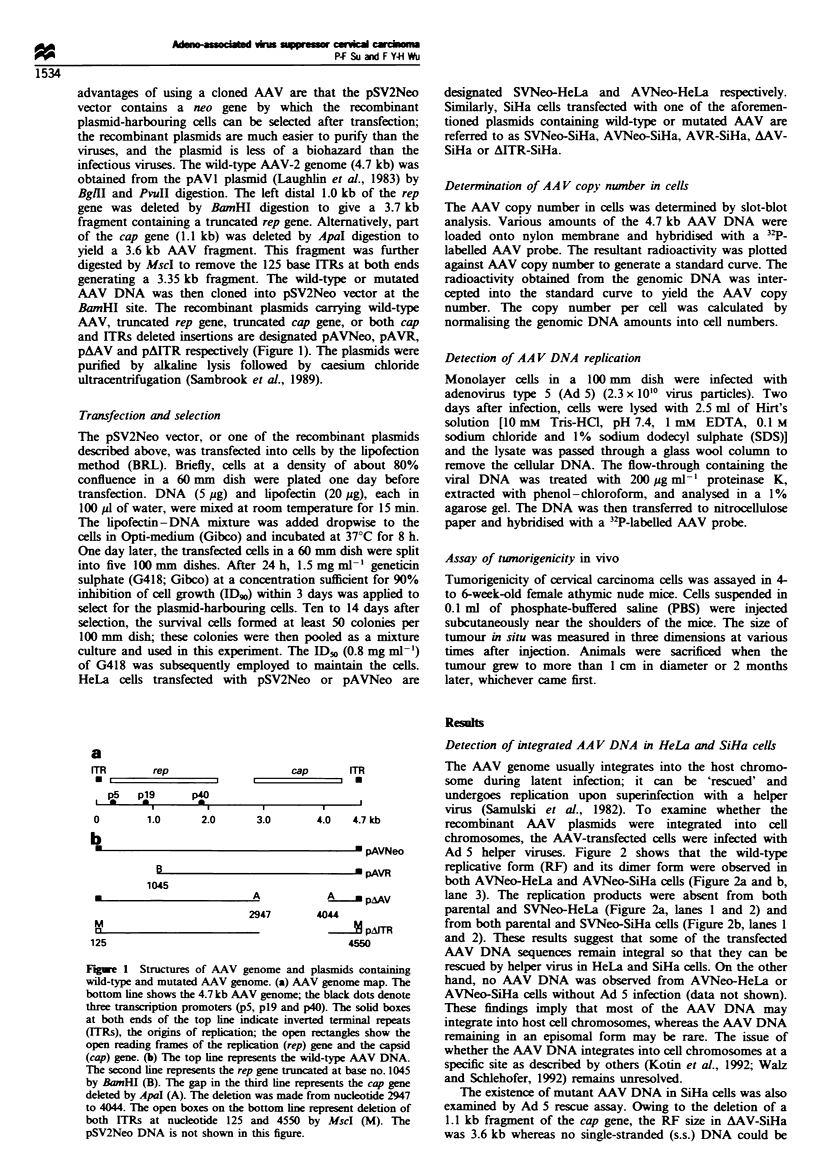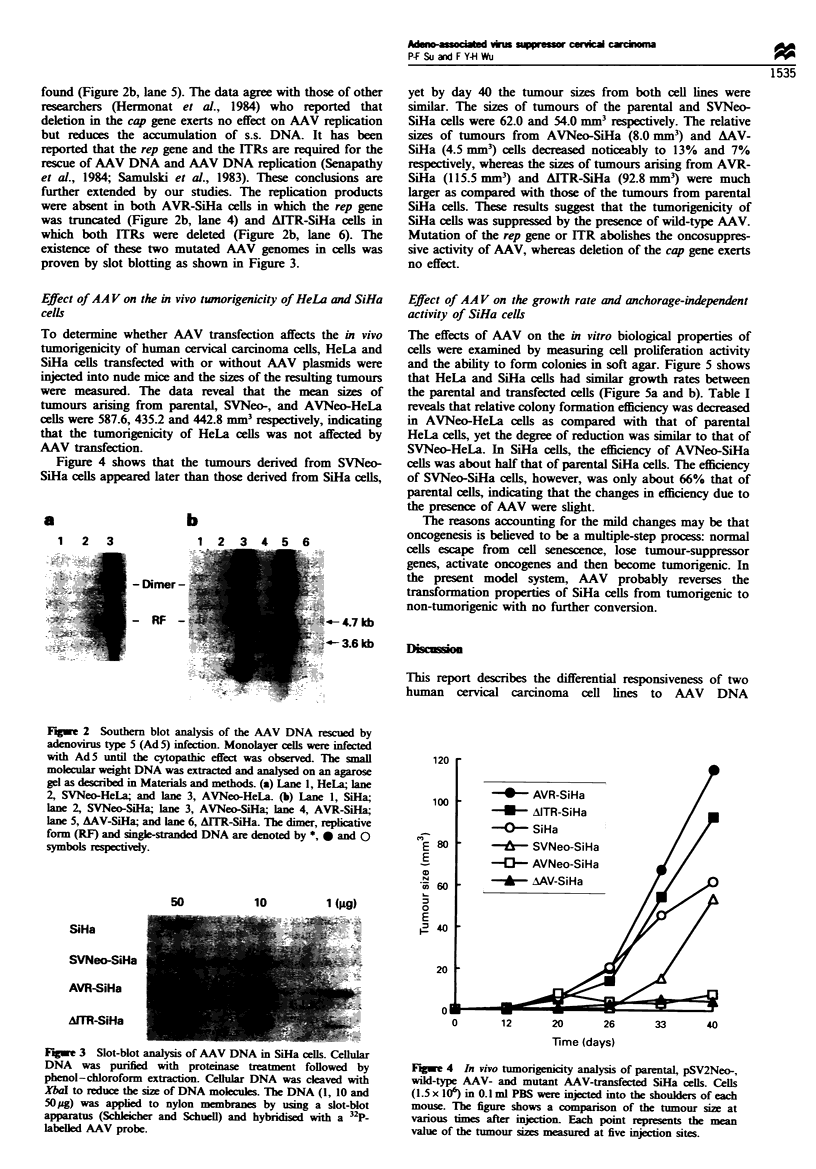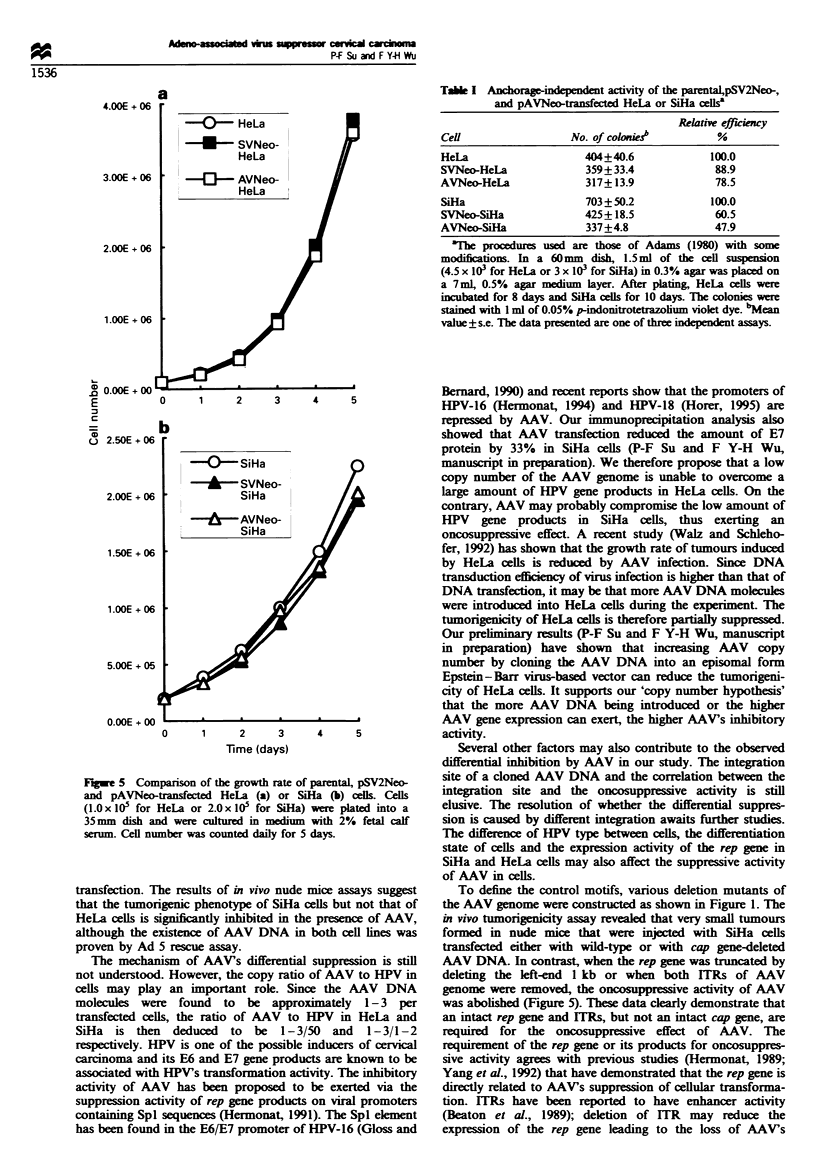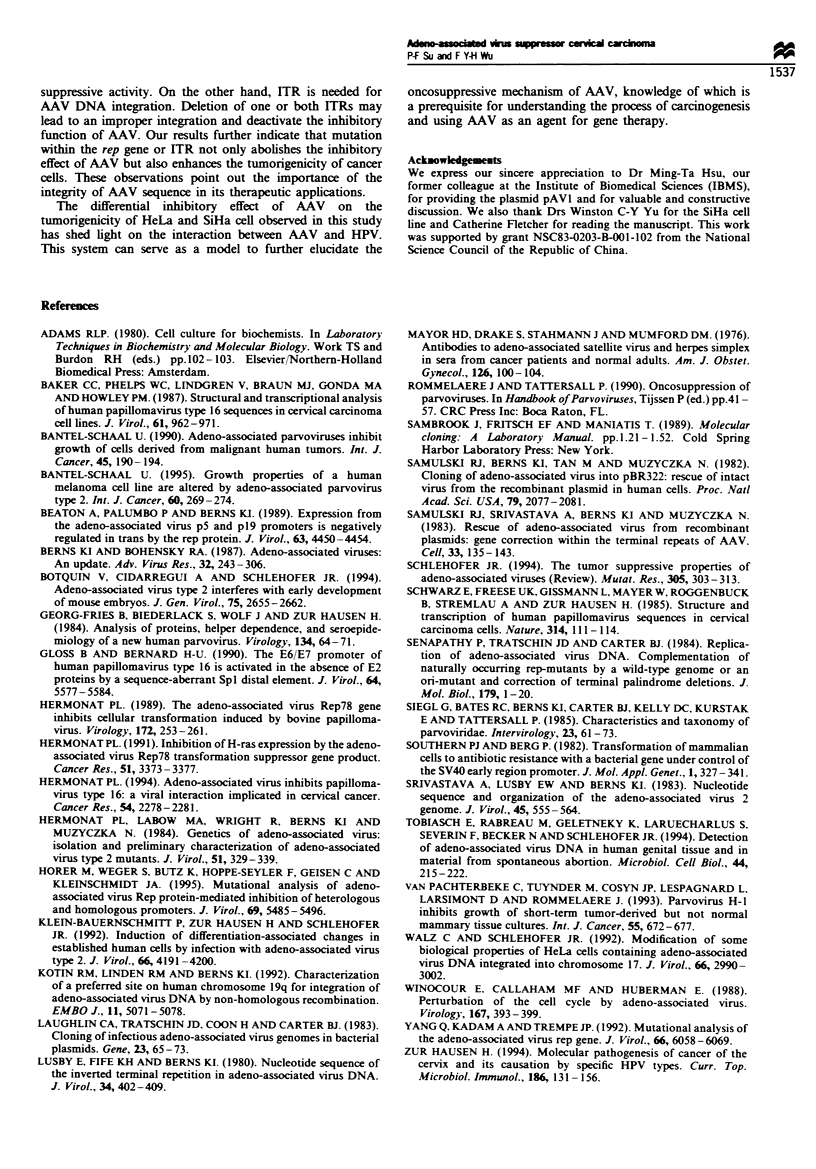# Differential suppression of the tumorigenicity of HeLa and SiHa cells by adeno-associated virus.

**DOI:** 10.1038/bjc.1996.289

**Published:** 1996-06

**Authors:** P. F. Su, F. Y. Wu

**Affiliations:** Division of Cancer Research, Institute of Biomedical Sciences, Academia Sinica, Taipei, Taiwan, Republic of China.

## Abstract

**Images:**


© 1996 Stockton Press All rights reserved 0007-0920/96 $12.00

### Differential suppression of the tumorigenicity of HeLa and SiHa cells by adeno-associated virus

P-F Su[1,2] and F Y-H Wu[1]

[1]*Division of Cancer Research, Institute of Biomedical Sciences, Academia Sinica, Taipei 115;* [2]*Graduate Institute of Life Sciences, National Defense Medical Center, Taipei 110, Taiwan, Republic of China.*

**Summary** Adeno-associated virus (AAV) is well known for suppression of oncogenesis in rodents, but its inhibitory effects on human carcinoma are less well understood. We report the differential ability of AAV to inhibit the tumorigenicity of two human cervical carcinoma cell lines. The wild-type AAV-2 DNA carried by a pSV2Neo vector was transfected into HeLa cells, which contain 50 copies of human papillomavirus type 18 (HPV-18), and SiHa cells, which contain 1-2 copies of HPV-16. About 1-3 copies of AAV genome were introduced per cell. AAV transfection moderately reduced the growth rate and anchorage-independent activity of the cells. In nude mice, the size of tumours arising from SiHa cells was reduced by 87%, in contrast to no reduction in tumour size arising from HeLa cells. This suggests that the differential suppression exerted by AAV may be due to differences in HPV copy number. To define the region that is responsible for the oncosuppression, mutation analyses were conducted. The results of nude mice assays showed that both the replication gene and inverted terminal repeats of AAV were important for the inhibition. This study may provide a model system for further studies on the underlying mechanism of AAV oncosuppressive activity.

**Keywords:** adeno-associated virus; human papillomavirus; human cervical carcinoma cell lines; oncosuppression; nude mice

The parvoviridae family has been divided into three genera based on the requirement for helper viruses. Autonomous parvovirus and densovirus replicate autonomously, whereas dependovirus needs a helper virus for replication (Siegl *et al.*, 1985). Autonomous virus and dependovirus interfere with both spontaneous oncogenesis and experiment-induced tumours in rodents (Rommelaere and Tattersall, 1990). The suppression is probably exerted through indirect, e.g. immune system or direct virus-host interactions (van Pachterbeke *et al.*, 1993). The underlying mechanism is however still elusive.

Adeno-associated virus (AAV), a dependovirus, significantly inhibits carcinogenesis induced by adenovirus (Ad), herpes simplex virus, bovine papillomavirus (BPV) and the *ras* oncogene in rodents (Schlehofer, 1994). In humans, no known disease has been associated with AAV infection (Berns and Bohensky, 1987), although recent reports have shown a linkage of AAV infection to miscarriage in mice and humans (Botquin *et al.*, 1994; Tobiasch *et al.*, 1994).

Studies of the effects of AAV on human cancer cells have led to four main conclusions. (1) AAV infection reduces human tumour cell proliferation (Bantel-Schaal, 1990), disturbs the cell cycle (Winocour *et al.*, 1988) and induces cell differentiation (Bantel-Schaal, 1995; Klein-Bauernschmitt *et al.*, 1992). (2) Seroepidemiological studies have found that the incidence of AAV antibody are lower in cervical or prostate carcinoma patients than in controls (Georg-Fries *et al.*, 1984; Mayor *et al.*, 1976). (3) AAV infection has been reported to suppress the promoter activity of human papillomavirus type 16 (HPV-16) (Hermonat, 1994), while HPV is one of the potent inducers of cervical carcinoma (zur Hausen, 1994). (4) AAV infection inhibits *in vitro* cellular transformation of rodent cells mediated by BPV, which is a close relative to HPV (Hermonat, 1989). Taken together, it seems that AAV infection is reciprocally related to human cervical carcinoma. However, the inhibitory activity of AAV in human cells is less well understood than rodents. Nude mice assay has shown that the tumour growth rate induced by human cervical carcinoma HeLa cells was moderately reduced by AAV infection (Walz and Schlehofer, 1992). The issue of whether AAV can suppress the tumorigenicity of human cervical carcinoma cells was thus pursued in this study.

The human AAV type 2 DNA was cloned into a pSV2Neo vector (Southern and Berg, 1982) to generate a pAVNeo plasmid. The pSV2Neo or pAVNeo DNA was transfected into two cervical carcinoma cell lines, HeLa and SiHa. HeLa contains 10-50 copies of HPV-18 DNA (Schwarz *et al.*, 1985) per cell and SiHa contains 1-2 copies of HPV-16 DNA (Baker *et al.*, 1987) per cell. The cells were selected by geneticin and expanded without cloning to avoid possible misleading conclusions derived from using a particular cloned cell. A mixed culture is also more similar to the *in vivo* condition than a cloned one. To assess whether the tumorigenicity of carcinoma cells can be affected by AAV transfection, a nude mouse assay was performed. The *in vitro* transformation properties including growth rate and anchorage-independent activity were compared in cells transfected with or without AAV. The AAV genome consists of a replication (*rep*) gene, a capsid (*cap*) gene (Srivastava *et al.*, 1983) and two palindromic inverted terminal repeats (ITRs) (Lusby *et al.*, 1980). To understand the control region for the oncosuppressive activity, the AAV genome was mutated. The data indicate that the *rep* gene and ITRs of AAV but not the *cap* gene are important for AAV's inhibitory activity.

### Materials and methods

### Cells

Human cervical carcinoma cell lines, HeLa and SiHa, were maintained in Dulbecco's modified Eagle medium (DMEM) and Earle's minimum essential medium (EMEM), respectively. All media were supplemented with 10% heat-inactivated (56°C, 30 min) fetal calf serum, non-essential amino acids, 0.03% L-glutamine and 50 $\mu g\ ml^{-1}$ gentamycin.

### Plasmids

In this experiment, the AAV DNA was introduced into cells in a recombinant plasmid form instead of virus. The

Correspondence: FY-H Wu, Division of Cancer Research, Institute of Biomedical Sciences, Academia Sinica, 128 Yen-Chiu-Yuan Road, Section II, Taipei 115, Taiwan, Republic of China.
Received 13 September 1995; revised 11 December 1995; accepted 20 December 1995

advantages of using a cloned AAV are that the pSV2Neo vector contains a *neo* gene by which the recombinant plasmid-harbouring cells can be selected after transfection; the recombinant plasmids are much easier to purify than the viruses, and the plasmid is less of a biohazard than the infectious viruses. The wild-type AAV-2 genome (4.7 kb) was obtained from the pAV1 plasmid (Laughlin *et al.*, 1983) by *BglII* and *PvuII* digestion. The left distal 1.0 kb of the *rep* gene was deleted by *BamHI* digestion to give a 3.7 kb fragment containing a truncated *rep* gene. Alternatively, part of the *cap* gene (1.1 kb) was deleted by *ApaI* digestion to yield a 3.6 kb AAV fragment. This fragment was further digested by *MscI* to remove the 125 base ITRs at both ends generating a 3.35 kb fragment. The wild-type or mutated AAV DNA was then cloned into pSV2Neo vector at the *BamHI* site. The recombinant plasmids carrying wild-type AAV, truncated *rep* gene, truncated *cap* gene, or both *cap* and ITRs deleted insertions are designated pAVNeo, pAVR, pΔAV and pΔITR respectively (Figure 1). The plasmids were purified by alkaline lysis followed by caesium chloride ultracentrifugation (Sambrook *et al.*, 1989).

*Transfection and selection*

The pSV2Neo vector, or one of the recombinant plasmids described above, was transfected into cells by the lipofection method (BRL). Briefly, cells at a density of about 80% confluence in a 60 mm dish were plated one day before transfection. DNA (5 μg) and lipofectin (20 μg), each in 100 μl of water, were mixed at room temperature for 15 min. The lipofectin–DNA mixture was added dropwise to the cells in Opti-medium (Gibco) and incubated at 37°C for 8 h. One day later, the transfected cells in a 60 mm dish were split into five 100 mm dishes. After 24 h, 1.5 mg ml$^{-1}$ geneticin sulphate (G418; Gibco) at a concentration sufficient for 90% inhibition of cell growth ($ID_{90}$) within 3 days was applied to select for the plasmid-harbouring cells. Ten to 14 days after selection, the survival cells formed at least 50 colonies per 100 mm dish; these colonies were then pooled as a mixture culture and used in this experiment. The $ID_{50}$ (0.8 mg ml$^{-1}$) of G418 was subsequently employed to maintain the cells. HeLa cells transfected with pSV2Neo or pAVNeo are designated SVNeo-HeLa and AVNeo-HeLa respectively. Similarly, SiHa cells transfected with one of the aforementioned plasmids containing wild-type or mutated AAV are referred to as SVNeo-SiHa, AVNeo-SiHa, AVR-SiHa, ΔAV-SiHa or ΔITR-SiHa.

*Determination of AAV copy number in cells*

The AAV copy number in cells was determined by slot-blot analysis. Various amounts of the 4.7 kb AAV DNA were loaded onto nylon membrane and hybridised with a $^{32}$P-labelled AAV probe. The resultant radioactivity was plotted against AAV copy number to generate a standard curve. The radioactivity obtained from the genomic DNA was intercepted into the standard curve to yield the AAV copy number. The copy number per cell was calculated by normalising the genomic DNA amounts into cell numbers.

*Detection of AAV DNA replication*

Monolayer cells in a 100 mm dish were infected with adenovirus type 5 (Ad 5) ($2.3 \times 10^{10}$ virus particles). Two days after infection, cells were lysed with 2.5 ml of Hirt's solution [10 mM Tris-HCl, pH 7.4, 1 mM EDTA, 0.1 M sodium chloride and 1% sodium dodecyl sulphate (SDS)] and the lysate was passed through a glass wool column to remove the cellular DNA. The flow-through containing the viral DNA was treated with 200 μg ml$^{-1}$ proteinase K, extracted with phenol–chloroform, and analysed in a 1% agarose gel. The DNA was then transferred to nitrocellulose paper and hybridised with a $^{32}$P-labelled AAV probe.

*Assay of tumorigenicity* in vivo

Tumorigenicity of cervical carcinoma cells was assayed in 4- to 6-week-old female athymic nude mice. Cells suspended in 0.1 ml of phosphate-buffered saline (PBS) were injected subcutaneously near the shoulders of the mice. The size of tumour *in situ* was measured in three dimensions at various times after injection. Animals were sacrificed when the tumour grew to more than 1 cm in diameter or 2 months later, whichever came first.

**Figure 1** Structures of AAV genome and plasmids containing wild-type and mutated AAV genome. (**a**) AAV genome map. The bottom line shows the 4.7 kb AAV genome; the black dots denote three transcription promoters (p5, p19 and p40). The solid boxes at both ends of the top line indicate inverted terminal repeats (ITRs), the origins of replication; the open rectangles show the open reading frames of the replication (*rep*) gene and the capsid (*cap*) gene. (**b**) The top line represents the wild-type AAV DNA. The second line represents the *rep* gene truncated at base no. 1045 by *BamHI* (B). The gap in the third line represents the *cap* gene deleted by *ApaI* (A). The deletion was made from nucleotide 2947 to 4044. The open boxes on the bottom line represent deletion of both ITRs at nucleotide 125 and 4550 by *MscI* (M). The pSV2Neo DNA is not shown in this figure.

### Results

*Detection of integrated AAV DNA in HeLa and SiHa cells*

The AAV genome usually integrates into the host chromosome during latent infection; it can be 'rescued' and undergoes replication upon superinfection with a helper virus (Samulski *et al.*, 1982). To examine whether the recombinant AAV plasmids were integrated into cell chromosomes, the AAV-transfected cells were infected with Ad 5 helper viruses. Figure 2 shows that the wild-type replicative form (RF) and its dimer form were observed in both AVNeo-HeLa and AVNeo-SiHa cells (Figure 2a and b, lane 3). The replication products were absent from both parental and SVNeo-HeLa (Figure 2a, lanes 1 and 2) and from both parental and SVNeo-SiHa cells (Figure 2b, lanes 1 and 2). These results suggest that some of the transfected AAV DNA sequences remain integral so that they can be rescued by helper virus in HeLa and SiHa cells. On the other hand, no AAV DNA was observed from AVNeo-HeLa or AVNeo-SiHa cells without Ad 5 infection (data not shown). These findings imply that most of the AAV DNA may integrate into host cell chromosomes, whereas the AAV DNA remaining in an episomal form may be rare. The issue of whether the AAV DNA integrates into cell chromosomes at a specific site as described by others (Kotin *et al.*, 1992; Walz and Schlehofer, 1992) remains unresolved.

The existence of mutant AAV DNA in SiHa cells was also examined by Ad 5 rescue assay. Owing to the deletion of a 1.1 kb fragment of the *cap* gene, the RF size in ΔAV-SiHa was 3.6 kb whereas no single-stranded (s.s.) DNA could be found (Figure 2b, lane 5). The data agree with those of other researchers (Hermonat *et al.*, 1984) who reported that deletion in the *cap* gene exerts no effect on AAV replication but reduces the accumulation of s.s. DNA. It has been reported that the *rep* gene and the ITRs are required for the rescue of AAV DNA and AAV DNA replication (Senapathy *et al.*, 1984; Samulski *et al.*, 1983). These conclusions are further extended by our studies. The replication products were absent in both AVR-SiHa cells in which the *rep* gene was truncated (Figure 2b, lane 4) and ΔITR-SiHa cells in which both ITRs were deleted (Figure 2b, lane 6). The existence of these two mutated AAV genomes in cells was proven by slot blotting as shown in Figure 3.

*Effect of AAV on the in vivo tumorigenicity of HeLa and SiHa cells*

To determine whether AAV transfection affects the *in vivo* tumorigenicity of human cervical carcinoma cells, HeLa and SiHa cells transfected with or without AAV plasmids were injected into nude mice and the sizes of the resulting tumours were measured. The data reveal that the mean sizes of tumours arising from parental, SVNeo-, and AVNeo-HeLa cells were 587.6, 435.2 and 442.8 mm$^3$ respectively, indicating that the tumorigenicity of HeLa cells was not affected by AAV transfection.

Figure 4 shows that the tumours derived from SVNeo-SiHa cells appeared later than those derived from SiHa cells, yet by day 40 the tumour sizes from both cell lines were similar. The sizes of tumours of the parental and SVNeo-SiHa cells were 62.0 and 54.0 mm$^3$ respectively. The relative sizes of tumours from AVNeo-SiHa (8.0 mm$^3$) and ΔAV-SiHa (4.5 mm$^3$) cells decreased noticeably to 13% and 7% respectively, whereas the sizes of tumours arising from AVR-SiHa (115.5 mm$^3$) and ΔITR-SiHa (92.8 mm$^3$) were much larger as compared with those of the tumours from parental SiHa cells. These results suggest that the tumorigenicity of SiHa cells was suppressed by the presence of wild-type AAV. Mutation of the *rep* gene or ITR abolishes the oncosuppressive activity of AAV, whereas deletion of the *cap* gene exerts no effect.

*Effect of AAV on the growth rate and anchorage-independent activity of SiHa cells*

The effects of AAV on the *in vitro* biological properties of cells were examined by measuring cell proliferation activity and the ability to form colonies in soft agar. Figure 5 shows that HeLa and SiHa cells had similar growth rates between the parental and transfected cells (Figure 5a and b). Table I reveals that relative colony formation efficiency was decreased in AVNeo-HeLa cells as compared with that of parental HeLa cells, yet the degree of reduction was similar to that of SVNeo-HeLa. In SiHa cells, the efficiency of AVNeo-SiHa cells was about half that of parental SiHa cells. The efficiency of SVNeo-SiHa cells, however, was only about 66% that of parental cells, indicating that the changes in efficiency due to the presence of AAV were slight.

The reasons accounting for the mild changes may be that oncogenesis is believed to be a multiple-step process: normal cells escape from cell senescence, lose tumour-suppressor genes, activate oncogenes and then become tumorigenic. In the present model system, AAV probably reverses the transformation properties of SiHa cells from tumorigenic to non-tumorigenic with no further conversion.

### Discussion

This report describes the differential responsiveness of two human cervical carcinoma cell lines to AAV DNA

**Figure 2** Southern blot analysis of the AAV DNA rescued by adenovirus type 5 (Ad 5) infection. Monolayer cells were infected with Ad 5 until the cytopathic effect was observed. The small molecular weight DNA was extracted and analysed on an agarose gel as described in Materials and methods. (**a**) Lane 1, HeLa; lane 2, SVNeo-HeLa; and lane 3, AVNeo-HeLa. (**b**) Lane 1, SiHa; lane 2, SVNeo-SiHa; lane 3, AVNeo-SiHa; lane 4, AVR-SiHa; lane 5, ΔAV-SiHa; and lane 6, ΔITR-SiHa. The dimer, replicative form (RF) and single-stranded DNA are denoted by *, ● and ○ symbols respectively.

**Figure 3** Slot-blot analysis of AAV DNA in SiHa cells. Cellular DNA was purified with proteinase treatment followed by phenol–chloroform extraction. Cellular DNA was cleaved with *Xba*I to reduce the size of DNA molecules. The DNA (1, 10 and 50 μg) was applied to nylon membranes by using a slot-blot apparatus (Schleicher and Schuell) and hybridised with a $^{32}$P-labelled AAV probe.

**Figure 4** *In vivo* tumorigenicity analysis of parental, pSV2Neo-, wild-type AAV- and mutant AAV-transfected SiHa cells. Cells ($1.5 \times 10^6$) in 0.1 ml PBS were injected into the shoulders of each mouse. The figure shows a comparison of the tumour size at various times after injection. Each point represents the mean value of the tumour sizes measured at five injection sites.

**Figure 5** Comparison of the growth rate of parental, pSV2Neo- and pAVNeo-transfected HeLa (**a**) or SiHa (**b**) cells. Cells ($1.0 \times 10^5$ for HeLa or $2.0 \times 10^5$ for SiHa) were plated into a 35 mm dish and were cultured in medium with 2% fetal calf serum. Cell number was counted daily for 5 days.

transfection. The results of *in vivo* nude mice assays suggest that the tumorigenic phenotype of SiHa cells but not that of HeLa cells is significantly inhibited in the presence of AAV, although the existence of AAV DNA in both cell lines was proven by Ad 5 rescue assay.

The mechanism of AAV's differential suppression is still not understood. However, the copy ratio of AAV to HPV in cells may play an important role. Since the AAV DNA molecules were found to be approximately 1-3 per transfected cells, the ratio of AAV to HPV in HeLa and SiHa is then deduced to be 1-3/50 and 1-3/1-2 respectively. HPV is one of the possible inducers of cervical carcinoma and its E6 and E7 gene products are known to be associated with HPV's transformation activity. The inhibitory activity of AAV has been proposed to be exerted via the suppression activity of *rep* gene products on viral promoters containing Spl sequences (Hermonat, 1991). The Spl element has been found in the E6/E7 promoter of HPV-16 (Gloss and

**Table I** Anchorage-independent activity of the parental, pSV2Neo-, and pAVNeo-transfected HeLa or SiHa cells[a]

| *Cell* | *No. of colonies*[b] | *Relative efficiency* % |
|---|---|---|
| HeLa | 404±40.6 | 100.0 |
| SVNeo-HeLa | 359±33.4 | 88.9 |
| AVNeo-HeLa | 317±13.9 | 78.5 |
| SiHa | 703±50.2 | 100.0 |
| SVNeo-SiHa | 425±18.5 | 60.5 |
| AVNeo-SiHa | 337±4.8 | 47.9 |

[a]The procedures used are those of Adams (1980) with some modifications. In a 60 mm dish, 1.5 ml of the cell suspension ($4.5 \times 10^3$ for HeLa or $3 \times 10^3$ for SiHa) in 0.3% agar was placed on a 7 ml, 0.5% agar medium layer. After plating, HeLa cells were incubated for 8 days and SiHa cells for 10 days. The colonies were stained with 1 ml of 0.05% *p*-indonitrotetrazolium violet dye. [b]Mean value±s.e. The data presented are one of three independent assays.

Bernard, 1990) and recent reports show that the promoters of HPV-16 (Hermonat, 1994) and HPV-18 (Horer, 1995) are repressed by AAV. Our immunoprecipitation analysis also showed that AAV transfection reduced the amount of E7 protein by 33% in SiHa cells (P-F Su and F Y-H Wu, manuscript in preparation). We therefore propose that a low copy number of the AAV genome is unable to overcome a large amount of HPV gene products in HeLa cells. On the contrary, AAV may probably compromise the low amount of HPV gene products in SiHa cells, thus exerting an oncosuppressive effect. A recent study (Walz and Schlehofer, 1992) has shown that the growth rate of tumours induced by HeLa cells is reduced by AAV infection. Since DNA transduction efficiency of virus infection is higher than that of DNA transfection, it may be that more AAV DNA molecules were introduced into HeLa cells during the experiment. The tumorigenicity of HeLa cells is therefore partially suppressed. Our preliminary results (P-F Su and F Y-H Wu, manuscript in preparation) have shown that increasing AAV copy number by cloning the AAV DNA into an episomal form Epstein-Barr virus-based vector can reduce the tumorigenicity of HeLa cells. It supports our 'copy number hypothesis' that the more AAV DNA being introduced or the higher AAV gene expression can exert, the higher AAV's inhibitory activity.

Several other factors may also contribute to the observed differential inhibition by AAV in our study. The integration site of a cloned AAV DNA and the correlation between the integration site and the oncosuppressive activity is still elusive. The resolution of whether the differential suppression is caused by different integration awaits further studies. The difference of HPV type between cells, the differentiation state of cells and the expression activity of the *rep* gene in SiHa and HeLa cells may also affect the suppressive activity of AAV in cells.

To define the control motifs, various deletion mutants of the AAV genome were constructed as shown in Figure 1. The *in vivo* tumorigenicity assay revealed that very small tumours formed in nude mice that were injected with SiHa cells transfected either with wild-type or with *cap* gene-deleted AAV DNA. In contrast, when the *rep* gene was truncated by deleting the left-end 1 kb or when both ITRs of AAV genome were removed, the oncosuppressive activity of AAV was abolished (Figure 5). These data clearly demonstrate that an intact *rep* gene and ITRs, but not an intact *cap* gene, are required for the oncosuppressive effect of AAV. The requirement of the *rep* gene or its products for oncosuppressive activity agrees with previous studies (Hermonat, 1989; Yang *et al*., 1992) that have demonstrated that the *rep* gene is directly related to AAV's suppression of cellular transformation. ITRs have been reported to have enhancer activity (Beaton *et al*., 1989); deletion of ITR may reduce the expression of the *rep* gene leading to the loss of AAV's suppressive activity. On the other hand, ITR is needed for AAV DNA integration. Deletion of one or both ITRs may lead to an improper integration and deactivate the inhibitory function of AAV. Our results further indicate that mutation within the *rep* gene or ITR not only abolishes the inhibitory effect of AAV but also enhances the tumorigenicity of cancer cells. These observations point out the importance of the integrity of AAV sequence in its therapeutic applications.

The differential inhibitory effect of AAV on the tumorigenicity of HeLa and SiHa cell observed in this study has shed light on the interaction between AAV and HPV. This system can serve as a model to further elucidate the oncosuppressive mechanism of AAV, knowledge of which is a prerequisite for understanding the process of carcinogenesis and using AAV as an agent for gene therapy.

**Acknowledgements**
We express our sincere appreciation to Dr Ming-Ta Hsu, our former colleague at the Institute of Biomedical Sciences (IBMS), for providing the plasmid pAV1 and for valuable and constructive discussion. We also thank Drs Winston C-Y Yu for the SiHa cell line and Catherine Fletcher for reading the manuscript. This work was supported by grant NSC83-0203-B-001-102 from the National Science Council of the Republic of China.

### References

ADAMS RLP. (1980). Cell culture for biochemists. In *Laboratory Techniques in Biochemistry and Molecular Biology*. Work TS and Burdon RH (eds.) pp.102-103. Elsevier/Northern-Holland Biomedical Press: Amsterdam.

BAKER CC, PHELPS WC, LINDGREN V, BRAUN MJ, GONDA MA AND HOWLEY PM. (1987). Structural and transcriptional analysis of human papillomavirus type 16 sequences in cervical carcinoma cell lines. *J. Virol.*, **61**, 962-971.

BANTEL-SCHAAL U. (1990). Adeno-associated parvoviruses inhibit growth of cells derived from malignant human tumors. *Int. J. Cancer*, **45**, 190-194.

BANTEL-SCHAAL U. (1995). Growth properties of a human melanoma cell line are altered by adeno-associated parvovirus type 2. *Int. J. Cancer*, **60**, 269-274.

BEATON A, PALUMBO P AND BERNS KI. (1989). Expression from the adeno-associated virus p5 and p19 promoters is negatively regulated in trans by the rep protein. *J. Virol.*, **63**, 4450-4454.

BERNS KI AND BOHENSKY RA. (1987). Adeno-associated viruses: An update. *Adv. Virus Res.*, **32**, 243-306.

BOTQUIN V, CIDARREGUI A AND SCHLEHOFER JR. (1994). Adeno-associated virus type 2 interferes with early development of mouse embryos. *J. Gen. Virol.*, **75**, 2655-2662.

GEORG-FRIES B, BIEDERLACK S, WOLF J AND ZUR HAUSEN H. (1984). Analysis of proteins, helper dependence, and seroepidemiology of a new human parvovirus. *Virology*, **134**, 64-71.

GLOSS B AND BERNARD H-U. (1990). The E6/E7 promoter of human papillomavirus type 16 is activated in the absence of E2 proteins by a sequence-aberrant Sp1 distal element. *J. Virol.*, **64**, 5577-5584.

HERMONAT PL. (1989). The adeno-associated virus Rep78 gene inhibits cellular transformation induced by bovine papillomavirus. *Virology*, **172**, 253-261.

HERMONAT PL. (1991). Inhibition of H-ras expression by the adeno-associated virus Rep78 transformation suppressor gene product. *Cancer Res.*, **51**, 3373-3377.

HERMONAT PL. (1994). Adeno-associated virus inhibits papillomavirus type 16: a viral interaction implicated in cervical cancer. *Cancer Res.*, **54**, 2278-2281.

HERMONAT PL, LABOW MA, WRIGHT R, BERNS KI AND MUZYCZKA N. (1984). Genetics of adeno-associated virus: isolation and preliminary characterization of adeno-associated virus type 2 mutants. *J. Virol.*, **51**, 329-339.

HORER M, WEGER S, BUTZ K, HOPPE-SEYLER F, GEISEN C AND KLEINSCHMIDT JA. (1995). Mutational analysis of adeno-associated virus Rep protein-mediated inhibition of heterologous and homologous promoters. *J. Virol.*, **69**, 5485-5496.

KLEIN-BAUERNSCHMITT P, ZUR HAUSEN H AND SCHLEHOFER JR. (1992). Induction of differentiation-associated changes in established human cells by infection with adeno-associated virus type 2. *J. Virol.*, **66**, 4191-4200.

KOTIN RM, LINDEN RM AND BERNS KI. (1992). Characterization of a preferred site on human chromosome 19q for integration of adeno-associated virus DNA by non-homologous recombination. *EMBO J.*, **11**, 5071-5078.

LAUGHLIN CA, TRATSCHIN JD, COON H AND CARTER BJ. (1983). Cloning of infectious adeno-associated virus genomes in bacterial plasmids. *Gene*, **23**, 65-73.

LUSBY E, FIFE KH AND BERNS KI. (1980). Nucleotide sequence of the inverted terminal repetition in adeno-associated virus DNA. *J. Virol.*, **34**, 402-409.

MAYOR HD, DRAKE S, STAHMANN J AND MUMFORD DM. (1976). Antibodies to adeno-associated satellite virus and herpes simplex in sera from cancer patients and normal adults. *Am. J. Obstet. Gynecol.*, **126**, 100-104.

ROMMELAERE J AND TATTERSALL P. (1990). Oncosuppression of parvoviruses. In *Handbook of Parvoviruses*, Tijssen P (ed.) pp.41-57. CRC Press Inc: Boca Raton, FL.

SAMBROOK J, FRITSCH EF AND MANIATIS T. (1989). *Molecular cloning: A Laboratory Manual*. pp.1.21-1.52. Cold Spring Harbor Laboratory Press: New York.

SAMULSKI RJ, BERNS KI, TAN M AND MUZYCZKA N. (1982). Cloning of adeno-associated virus into pBR322: rescue of intact virus from the recombinant plasmid in human cells. *Proc. Natl Acad. Sci. USA*, **79**, 2077-2081.

SAMULSKI RJ, SRIVASTAVA A, BERNS KI AND MUZYCZKA N. (1983). Rescue of adeno-associated virus from recombinant plasmids: gene correction within the terminal repeats of AAV. *Cell*, **33**, 135-143.

SCHLEHOFER JR. (1994). The tumor suppressive properties of adeno-associated viruses (Review). *Mutat. Res.*, **305**, 303-313.

SCHWARZ E, FREESE UK, GISSMANN L, MAYER W, ROGGENBUCK B, STREMLAU A AND ZUR HAUSEN H. (1985). Structure and transcription of human papillomavirus sequences in cervical carcinoma cells. *Nature*, **314**, 111-114.

SENAPATHY P, TRATSCHIN JD AND CARTER BJ. (1984). Replication of adeno-associated virus DNA. Complementation of naturally occurring rep-mutants by a wild-type genome or an ori-mutant and correction of terminal palindrome deletions. *J. Mol. Biol.*, **179**, 1-20.

SIEGL G, BATES RC, BERNS KI, CARTER BJ, KELLY DC, KURSTAK E AND TATTERSALL P. (1985). Characteristics and taxonomy of parvoviridae. *Intervirology*, **23**, 61-73.

SOUTHERN PJ AND BERG P. (1982). Transformation of mammalian cells to antibiotic resistance with a bacterial gene under control of the SV40 early region promoter. *J. Mol. Appl. Genet.*, **1**, 327-341.

SRIVASTAVA A, LUSBY EW AND BERNS KI. (1983). Nucleotide sequence and organization of the adeno-associated virus 2 genome. *J. Virol.*, **45**, 555-564.

TOBIASCH E, RABREAU M, GELETNEKY K, LARUECHARLUS S, SEVERIN F, BECKER N AND SCHLEHOFER JR. (1994). Detection of adeno-associated virus DNA in human genital tissue and in material from spontaneous abortion. *Microbiol. Cell Biol.*, **44**, 215-222.

VAN PACHTERBEKE C, TUYNDER M, COSYN JP, LESPAGNARD L, LARSIMONT D AND ROMMELAERE J. (1993). Parvovirus H-1 inhibits growth of short-term tumor-derived but not normal mammary tissue cultures. *Int. J. Cancer*, **55**, 672-677.

WALZ C AND SCHLEHOFER JR. (1992). Modification of some biological properties of HeLa cells containing adeno-associated virus DNA integrated into chromosome 17. *J. Virol.*, **66**, 2990-3002.

WINOCOUR E, CALLAHAM MF AND HUBERMAN E. (1988). Perturbation of the cell cycle by adeno-associated virus. *Virology*, **167**, 393-399.

YANG Q, KADAM A AND TREMPE JP. (1992). Mutational analysis of the adeno-associated virus rep gene. *J. Virol.*, **66**, 6058-6069.

ZUR HAUSEN H. (1994). Molecular pathogenesis of cancer of the cervix and its causation by specific HPV types. *Curr. Top. Microbiol. Immunol.*, **186**, 131-156.